# Glutamate–Serine–Glycine Index: A Novel Potential Biomarker in Pediatric Non-Alcoholic Fatty Liver Disease

**DOI:** 10.3390/children7120270

**Published:** 2020-12-04

**Authors:** Simone Leonetti, Raimund I. Herzog, Sonia Caprio, Nicola Santoro, Domenico Tricò

**Affiliations:** 1 Department of Surgical, Medical and Molecular Pathology and Critical Care Medicine, University of Pisa, 56126 Pisa, Italy; s.leonetti@live.com; 2 Department of Internal Medicine, Section of Endocrinology, Yale University School of Medicine, New Haven, CT 06510, USA; raimund.herzog@yale.edu; 3 Department of Pediatrics, Yale University School of Medicine, New Haven, CT 06510, USA; sonia.caprio@yale.edu (S.C.); nicola.santoro@yale.edu (N.S.); 4 Department of Medicine and Health Sciences, “V.Tiberio” University of Molise, 86100 Campobasso, Italy

**Keywords:** amino acids, non-alcoholic fatty liver disease, pediatric obesity, insulin resistance

## Abstract

Preliminary evidence suggests that the glutamate–serine–glycine (GSG) index, which combines three amino acids involved in glutathione synthesis, may be used as a potential biomarker of non-alcoholic fatty liver disease (NAFLD). We investigated whether the GSG index is associated with NAFLD in youth, independent of other risk factors. Intrahepatic fat content (HFF%) and abdominal fat distribution were measured by magnetic resonance imaging (MRI) in a multiethnic cohort of obese adolescents, including Caucasians, African Americans, and Hispanics. NAFLD was defined as HFF% ≥ 5.5%. Plasma amino acids were measured by mass spectrometry. The GSG index was calculated as glutamate/(serine + glycine). The GSG index was higher in NAFLD patients (*p* = 0.03) and positively correlated with HFF% (r = 0.26, *p* = 0.02), alanine aminotransferase (r = 0.39, *p* = 0.0006), and aspartate aminotransferase (r = 0.26, *p* = 0.03). Adolescents with a high GSG index had a twofold higher prevalence of NAFLD than those with a low GSG index, despite similar adiposity, abdominal fat distribution, and liver insulin resistance. NAFLD prevalence remained significantly different between groups after adjustment for age, sex, race/ethnicity, and body mass index (OR 3.07, 95% confidence interval 1.09–8.61, *p* = 0.03). This study demonstrates the ability of the GSG index to detect NAFLD in at-risk pediatric populations with different genetically determined susceptibilities to intrahepatic fat accumulation, independent of traditional risk factors.

## 1. Introduction

Non-alcoholic fatty liver disease (NAFLD) has become the most common chronic liver disease worldwide, with an estimated prevalence of 40% in obese youth [1]. Therefore, there is an urgent clinical need for non-invasive, high-throughput diagnostic tools for NAFLD. 

Metabolomics studies have recently identified specific amino acid patterns that may be used as potential biomarkers of liver disease [2]. Among them, Gaggini et al. [3] proposed a novel glutamate–serine–glycine (GSG) index, which combines three amino acids involved in glutathione synthesis and hepatic lipotoxicity. GSG index was found to be significantly higher in adults with biopsy-proven NAFLD compared with lean controls and was associated with liver enzymes and hepatic insulin resistance [3]. Furthermore, two preliminary reports showed that GSG index changes match longitudinal changes of surrogate markers of fatty liver after pharmacological or nutritional therapy [4,5]. These studies were limited by the lack of a control group of obese subjects without NAFLD [3,5] and of a direct quantification of liver fat content [4]. Moreover, the ability of GSG index to detect NAFLD has not been established in youths and different ethnic groups. 

This study investigated whether the GSG index is associated with intrahepatic fat content in youth, independent of other risk factors.

## 2. Methods

### 2.1. Study Participants

Seventy-eight obese adolescents, including 26 (33.3%) Caucasians, 22 (28.2%) African Americans, and 29 (37.2%) Hispanics, were recruited from the Yale Pediatric NAFLD study cohort (age 13.3 ± 3.0 years, 38 boys and 40 girls, body mass index (BMI) z-score 2.31 ± 0.34), a long-term project aimed at studying metabolic and metagenomic alterations in obese youths [1,6], as previously reported [7]. All participants had a detailed medical history and a complete physical examination. Participants’ ages ranged from 8–18 years and they had a BMI ≥95th percentile for age and sex. The main exclusion criteria were the use of medications known to affect liver function or amino acid metabolism; liver diseases besides NAFLD; and alcohol consumption. The study was approved by the Yale University Human Investigation Committee (HIC protocol #1104008388). All clinical investigations were conducted according to the principles expressed in the Declaration of Helsinki. Written informed parental consent and child assent were obtained from all participants.

### 2.2. Oral Glucose Tolerance Test

Participants were admitted to the Yale Center for Clinical Investigation (YCCI) at 8 a.m. after a 12-h overnight fast to undergo a 75 g oral glucose tolerance test (OGTT). One antecubital intravenous catheter was inserted for blood sampling after the local application of a topical anesthetic cream (Emla, Astra Zeneca, Wilmington, DE). Fasting blood samples were then obtained for measurements of plasma glucose, insulin, lipid profile, amino acids, and liver enzymes. Thereafter, flavored glucose in a dose of 1.75 g per kilogram of body weight (up to a maximum of 75 g) was given orally, and arterialized venous blood samples were obtained every 30 min for 180 min for the measurement of plasma glucose and insulin.

### 2.3. Magnetic Resonance Imaging

Quantification of the hepatic fat fraction (HFF%) was performed by liver magnetic resonance imaging (MRI) on a Siemens Sonata 1.5 Tesla system (Erlangen, Germany) using the two-point Dixon (2PD) method as modified by Fishbein et al. [8]. NAFLD was defined as HFF% ≥ 5.5% [7]. The 2PD method was previously validated in obese adolescents against liver biopsy [1] and proton nuclear magnetic resonance (NMR) [9]. Visceral and subcutaneous fat depots were quantified by abdominal MRI [10].

### 2.4. Biochemical Analysis

Glucose samples were spun immediately and processed at the bedside using a YSI2700-STAT-Analyzer (Yellow Springs Instruments, Yellow Springs, OH, USA). Plasma insulin was measured by radioimmunoassay (Linco, St. Charles, MO, USA) that has <1% cross-reactivity with C-peptide and proinsulin. Plasma amino acids were measured using AbsoluteIDQ mass spectrometry-based assay kits (Biocrates Institutes, Innsbruck, Austria), as previously reported [7].

### 2.5. Calculations

The GSG index was calculated as glutamate/(serine + glycine) according to Gaggini et al. [3]. The hepatic insulin resistance index (HIRI) was calculated as a product of the glucose area under the curve (AUC) and insulin AUC during the first 30 min of the OGTT [11]. The Matsuda index was used to estimate whole-body insulin sensitivity (Whole-Body Insulin Sensitivity Index, WBISI) [12]. The insulinogenic index (IGI), which represents early phase insulin secretion, was calculated as the ratio of insulin at 30 min minus fasting insulin to the difference in glucose at 30 min minus fasting glucose. The disposition index (DI), which provides an integrated picture of glucose tolerance, including both insulin sensitivity and insulin secretion, was calculated as the product of the IGI and the WBISI [13].

### 2.6. Statistical Analysis

Group differences were analyzed using Student’s *t*-test, Wilcoxon–Mann–Whitney test, or χ^2^ test, as appropriate. Correlations were tested by Spearman correlation. To account for potential confounders, multivariable regression analysis was used including age, sex, BMI z-score, and race as independent variables. Odds ratios (OR) and 95% confidence intervals (95%) from logistic regression analyses are reported. A cut point of 0.36 identified the upper tertile of GSG index distribution and was used to stratify subjects in GSG-high and GSG-low. Analyses were performed using JMP Pro 14 (SAS Institutes, Cary, NC, USA) at a two-sided α level of 0.05.

## 3. Results

The GSG index was higher in adolescents with NAFLD (0.34 [0.28–0.51]) than without NAFLD (0.29 [0.19–0.40], *p* = 0.03) but similar in boys and girls (*p* = 0.45) and among different ethnic groups (*p* = 0.40). The GSG index correlated directly with HFF% (r = 0.26, *p* = 0.02), alanine aminotransferase (ALT; r = 0.39, *p* = 0.0006), and aspartate aminotransferase (AST; r = 0.26, *p* = 0.03). No relationships were found between GSG index and age, BMI z-score, markers of glucose control, lipid profile, visceral and subcutaneous fat, branched-chain amino acids (BCAA), and HIRI (all *p* > 0.05). The effect of GSG index on NAFLD prevalence and HFF% remained significant in multivariable models adjusted for age, sex, BMI z-score, and race (β = 1.25, *p* = 0.01 and β = 1.07, *p* = 0.02, respectively).

**Figure 1:**
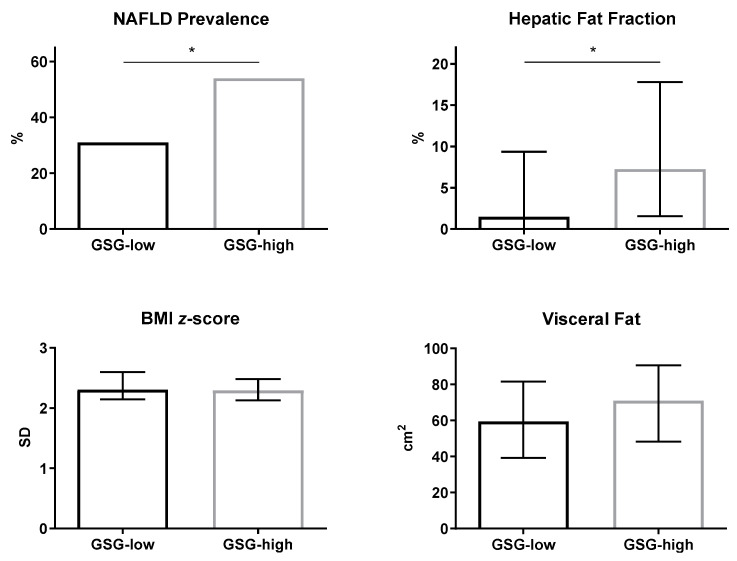
Prevalence of non-alcoholic fatty liver disease (NAFLD), hepatic fat fraction, BMI z-score, and visceral fat in obese adolescents with GSG-low and GSG-high. NAFLD prevalence is reported as percentage. Other data are reported as median and interquartile range. * *p* < 0.05.

The two groups of GSG-high and GSG-low subjects were matched for age, sex, ethnicity, adiposity, glucose control, and cholesterol profile (Table 1). The prevalence of NAFLD and HFF% were significantly higher in adolescents with GSG-high compared with GSG-low, despite similar BMI z-score and abdominal fat distribution (Figure 1). In multivariable logistic regression analysis, the likelihood of NAFLD increased in the GSG-high group (OR 3.07, 95%CI [1.09–8.61], *p* = 0.03) independent of age, sex, BMI z-score, and race.

## 4. Discussion

This study demonstrates the ability of the recently developed GSG index to detect NAFLD and its associated metabolic alterations (i.e., hypertriglyceridemia) in an at-risk population of obese adolescents. Noteworthy, by implementing an accurate MRI-based characterization of abdominal fat distribution, we observed for the first time that the relationship between GSG index and HFF% is independent of overall adiposity and visceral fat, supporting its role as a specific marker of intrahepatic fat content. This study also validates the use of the GSG index as a marker of NAFLD in pediatric populations and ethnic groups with different susceptibilities to intrahepatic fat accumulation, such as African Americans and Hispanics [1].

A recent untargeted, high-resolution metabolomics study identified several amino acid pathways dysregulated in adolescents with NAFLD, including glutamate, serine and glycine metabolism [14]. These amino acids are critical for the synthesis of glutathione, whose turnover is upregulated in NAFLD in response to oxidative stress, and of lipids associated with hepatic lipotoxicity (i.e., ceramides). Glycine and serine are consumed in these processes and are typically reduced in NAFLD [2]. Conversely, glutamate is consistently increased in NAFLD and other metabolic diseases [3]. In pathological conditions characterized by insulin resistance, such as NAFLD, obesity and metabolic syndrome, hepatic energy demands are increased due to altered mitochondrial metabolism and function [15,16,17]. In this context, the synthesis of liver aminotransferases is stimulated to cope with the increased need for transamination reactions [18]. Altered transaminase reactions, in turn, promote glutamate release and can justify the rise in plasma glutamate levels [19]. Furthermore, glutamate can be synthesized from proline metabolism. Higher proline concentrations have been reported in NAFLD in some [20], but not all [3], previous studies in adults. In our pediatric cohort, serum proline was similar in adolescents in the GSG-high and GSG-low group (*p* = 0.72), as well as in those with or without NAFLD (*p* = 0.14). Hence, the rise in glutamate is unlikely to be attributable to different proline concentrations. Finally, increased glutamate levels may also reflect greater dietary glutamate intake, which was not evaluated in our cohort.

The GSG index was initially associated with circulating liver enzymes and NAFLD histological severity [3], although subsequent biopsy studies do not support these findings [21]. In agreement with the earlier study, we found higher ALT and AST concentrations in the GSG-high group compared with the GSG-low group. Circulating transaminases are commonly used markers of hepatocellular damage, as they reflect the leaking of intracellular hepatocyte content into the circulation after liver injury. However, liver transaminase synthesis and release can also be enhanced in NAFLD as an adaptation to the increased metabolic demands, as mentioned above [18]. Altogether, these observations warrant further research to establish the GSG index as a reliable biomarker of NAFLD histological activity.

A marked increase in circulating branched-chain amino acids (BCAA) has been consistently reported in insulin-resistant states [22,23,24], including adult [3,25] and pediatric [7,14] NAFLD. Although an association between GSG index and total BCAA has been previously described [3], we could not find a significant relationship between GSG index and either single or total BCAA. This observation may be attributable to different study populations. Additionally, given that our data do not confirm the relation between GSG index and HIRI [3,4], it could be speculated that alterations in GSG index and BCAA differentially mark the distinct histological and metabolic features of NAFLD, namely inflammation/oxidative stress (for GSG index) and insulin resistance (for BCAA).

In conclusion, the GSG index represents a promising, minimally invasive biomarker for risk assessment of NAFLD in obese youths that is amenable to further validation in larger prospective cohort studies. These studies will provide an opportunity to establish whether the GSG index also correlates with NAFLD histological severity.

## Figures and Tables

**Table 1:** Characteristics of participants stratified by glutamate–serine–glycine (GSG) index—high and low.

	GSG High (*n* = 26)	GSG Low (*n* = 52)	*p*
CLINICAL FEATURES			
Age (years)	12.7 ± 3.0	13.6 ± 3.0	0.30
Sex (M/F)	16 (62%)/10 (38%)	22 (42%)/30 (58%)	0.11
Race (Caucasian/African American/ Hispanic/Asian)	8 (31%)/4 (15%)/14 (54%)/0 (0%)	18 (35%)/18 (35%)/15 (29%)/1 (1%)	0.12
Body Mass Index (kg/m^2^)	32.5 ± 6.9	34.69 ± 6.25	0.18
Body Mass Index z-score	2.29 ± 0.28	2.32 ± 0.37	0.73
Systolic blood pressure (mmHg)	115 ± 12	116 ± 9	0.66
Diastolic blood pressure (mmHg)	67 ± 8	68 ± 8	0.59
GLUCOSE METABOLISM			
Fasting glucose (mg/dL)	93 ± 7	92 ± 7	0.84
Fasting insulin (µU/mL)	27 [1.8–45]	27 [2.0–41]	0.71
2 h glucose (mg/dL)	120 ± 27	119 ± 24	0.86
Hemoglobin A1C (%)	5.52 ± 0.31	5.46 ± 0.30	0.41
HOMA-IR	6.23 [4.20–11.16]	6.51 [4.38–9.26]	0.66
Whole-Body Insulin Sensitivity Index	1.70 [0.74–2.46]	1.73 [1.11–2.39]	0.67
Hepatic Insulin Resistance Index	1611 [905–2403]	1499 [946–2018]	0.35
Insulinogenic Index	4.06 [2.43–6.50]	3.67 [2.85–5.08]	0.79
Disposition Index	5.05 [3.85–7.01]	5.88 [4.49–9.51]	0.34
LIPID PROFILE			
Total Cholesterol (mg/dL)	159 [140–179]	142 [131–169]	0.14
HDL Cholesterol (mg/dL)	40 [33–49]	46 [39–51]	0.07
LDL Cholesterol (mg/dL)	92 [76–119]	86 [72–102]	0.29
Triglycerides (mg/dL)	119 [85–140]	68 [50–106]	<0.0001
AMINO ACID PROFILE			
Glutamate (μmol/L)	147 [121–177]	73 [57–86]	<0.0001
Serine (μmol/L)	100 [88–106]	102 [87–117]	0.28
Glycine (μmol/L)	175 [149–203]	199 [180–239]	0.04
GSG index	0.50 [0.47–0.58]	0.25 [0.17–0.31]	<0.0001
BCAA (μmol/L)	413 [363–491]	423 [361–475]	0.70
ABDOMINAL FAT COMPOSITION			
Visceral (cm^2^)	70.9 [48.5–90.6]	59.4 [39.6–81]	0.24
Subcutaneous (cm^2^)	470.2 [356.2–680.2]	540.1 [398.8–734.1]	0.27
Visceral/Subcutaneous (%)	10.3 [8.7–14.9]	11.0 [8.2–16.7]	0.58
Visceral/Total (%)	9.4 [8.1–13.5]	10.0 [7.8–14.3]	0.67
LIVER ENZYMES			
Alanine transaminase (U/L)	24 [18–40]	17 [12–22]	0.001
Aspartate transaminase (U/L)	23 [20–32]	20 [17–25]	0.07

Data are number (percentage), mean ± SD, or median [interquartile range], as appropriate. Abbreviations: BCAA, branched-chain amino acids (i.e., isoleucine, leucine, and valine); HOMA-IR, homeostatic model assessment for insulin resistance.
